# Apolipoprotein C-III as a Potential Modulator of the Association Between HDL-Cholesterol and Incident Coronary Heart Disease

**DOI:** 10.1161/JAHA.111.000232

**Published:** 2012-04-24

**Authors:** Majken K. Jensen, Eric B. Rimm, Jeremy D. Furtado, Frank M. Sacks

**Affiliations:** Department of Nutrition, Harvard School of Public Health, Boston, MA (M.K.J., E.B.R., J.D.F., F.M.S.); Department of Epidemiology, Harvard School of Public Health, Boston, MA (E.B.R.); Channing Laboratory, Department of Medicine, Brigham and Women's Hospital, Harvard Medical School, Boston, MA (E.B.R., F.M.S.)

**Keywords:** apolipoproteins, cardiovascular disease, epidemiology, lipids

## Abstract

**Background:**

High-density lipoproteins (HDL) are structurally and metabolically heterogeneous and subclasses with differential effects on coronary heart disease (CHD) might exist. Apolipoprotein (apo) C-III, a small proinflammatory protein that resides on the surface of lipoproteins, enhances the atherogenicity of VLDL and LDL particles, but little is known about the role apoC-III on HDL. We investigated whether the presence or absence of apoC-III differentiates HDL into subtypes with nonprotective or protective associations with risk of future CHD.

**Methods and Results:**

High-density lipoprotein cholesterol (HDL-C) levels were measured in plasma separated according to apoC-III (by immunoaffinity chromatography) in two prospective case-control studies nested within the Nurses’ Health and the Health Professionals Follow-Up Studies. Baseline was in 1990 and 1994, and 634 incident CHD cases were documented through 10 to 14 years of follow-up. The relative risk of CHD per each standard deviation of total HDL-C was 0.78 (95% confidence intervals, 0.63–0.96). The HDL-C subtypes were differentially associated with risk of CHD, HDL-C without apoC-III inversely and HDL-C with apoC-III directly (*P*=0.02 for a difference between the HDL types). The relative risk per standard deviation of HDL-C without apoC-III was 0.66 (0.53 to 0.93) and 1.18 (1.03 to 1.34) for HDL-C with apoC-III. HDL-C with apoC-III comprised ∼13% of the total HDL-C. Adjustment for triglycerides and apoB attenuated the risks; however, the two HDL-C subgroups remained differentially associated with risk of CHD (*P*=0.05).

**Conclusion:**

Separating HDL-C according to apoC-III identified two types of HDL with opposing associations with risk of CHD. The proatherogenic effects of apoC-III, as a component of VLDL and LDL, may extend to HDL. **(*J Am Heart Assoc*. 2012;1:jah3-e000232 doi: 10.1161/JAHA.111.000232.)**

## Introduction

Population studies have shown that low-density lipoprotein cholesterol (LDL-C) directly and high-density lipoprotein cholesterol (HDL-C) inversely predict risk of coronary heart disease (CHD).^1–5^ Although statins and other classes of drugs efficiently reduce LDL-C and concomitantly lower the risk of cardiovascular events,^6^ evidence for independent atheroprotective effects of raising HDL-C is inconsistent.^7^ The anti-atherogenic properties of the HDL particle include the ability to promote transport of cholesterol from peripheral tissues such as the artery wall to the liver, as well as anti-inflammatory, anti-apoptotic, nitric oxide-promoting, prostacyclin-stabilizing, and platelet-inhibiting functions.^8^ However, changes in HDL-C among all trials using hypolipidemic drugs did not independently predict changes in CHD;^7,9^ and the lack of CHD reduction in trials of a drug that raises HDL-C by an unprecedented amount using a novel mechanism suggests the possibility that HDL-C may contain protective and nonprotective components.^10,11^

The metabolic heterogeneity of HDL particles may underlie the inconsistency between epidemiological studies, consistently showing independent risk prediction, and experimental approaches in clinical trials of lipid treatments. HDL comprises a diverse group of lipoproteins with substantial differences in size and density, and composition of lipids and proteins that influence the functional properties and metabolism of the particles. Thus, it is likely that subpopulations of HDL exist with more or less anti-atherogenic potential.^12–15^ Several large-scale epidemiologic studies have investigated the risk of CHD when HDL was separated by size. In some studies the concentration of small size HDL, or increase in small size HDL caused by gemfibrozil, is associated with lower incidence of CHD;^16–18^ in other studies, large size HDL had the protective associations;^19,20^ and in another cohort study, very large HDL particle size was directly associated with incidence of CAD.^21^ Thus, it remains inconclusive whether any of these techniques lead to any gain in information in terms of the identification of HDL subclasses with variable anti-atherogenic potential. Efforts to identify characteristics that may modulate the functional properties and metabolism of the HDL particle are important to improve the understanding of the atherosclerotic process and to prevent and treat cardiovascular diseases.

In previous work, we found that apolipoprotein (apo) C-III, a small protein that resides on the surface of some lipoproteins,^22,23^ provoked inflammatory and atherogenic responses in cells that are involved in atherosclerosis.^24,25^ The plasma concentration of apolipoprotein C-III (apoC-III) in VLDL and LDL, or the concentration of LDL that has apoC-III predicted risk of cardiovascular disease (CVD) or progression of coronary atherosclerosis independently of standard lipid risk factors.^26–29^ Although HDL particles exist both with and without apoC-III, little is known about the role of apoC-III in relation to HDL function or risk of CHD. Because apoC-III may inhibit anti-atherogenic actions of HDL,^25^ we aimed to compare plasma concentrations of total HDL, HDL that has apoC-III, and HDL without apoC-III as predictors of the risk of CHD in two prospective studies of US women and men initially free of CHD.

## Methods

### Design and Population

**Figure 1.**
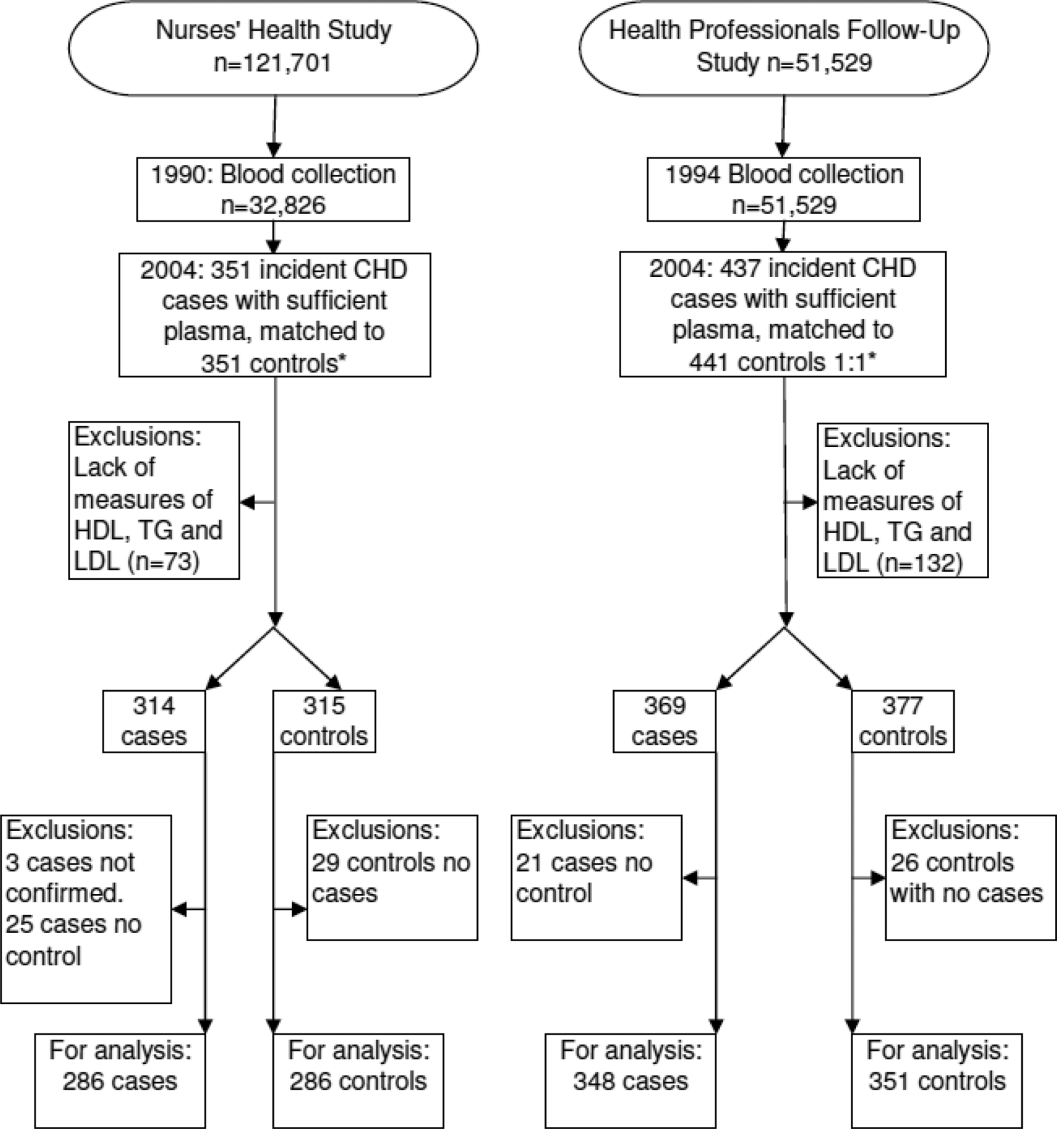
Flowchart for the nested case-control studies.

The Nurses' Health Study (NHS) enrolled 121 701 female nurses aged 30 to 55 years in 1976 and the Health Professionals Follow-Up Study (HPFS) enrolled 51 529 males aged 35 to 75 years in 1986. Participants of both cohorts filled out questionnaires on lifestyle and medical history and have since been followed with biennial questionnaires to record newly diagnosed illnesses and to update lifestyle information.^30,31^ Between 1989 and 1990, a blood sample was requested from all active participants in NHS and collected from 32 826 women. Similarly, blood samples were requested between 1993 and 1995 and obtained from 18 225 HPFS participants. As described earlier, nested case-control studies of CHD were designed within both cohorts, allowing the study to maintain a prospective design.^32^ Because the laboratory measurements for the present study required a large volume (0.600 mL), we restricted our study to those with more than 2 mL plasma (Figure 1). Among participants who were free of diagnosed cardiovascular disease or cancer at blood draw, we identified 351 women in NHS and 437 men in HPFS with incident CHD between blood draw and June, 2004. Using risk-set sampling,^33^ controls were selected randomly and matched in a 1:1 ratio on age (1 year), smoking (never, past, current), and month of blood return, among participants who were free of cardiovascular disease at the time CHD was diagnosed in the case. The diagnosis of CHD included nonfatal myocardial infarction and fatal CHD. The diagnosis of myocardial infarction was confirmed on the basis of the criteria of the World Health Organization (symptoms plus either diagnostic electrocardiographic changes or elevated levels of cardiac enzymes). Deaths were identified from state vital records and the National Death Index or reported by the participant's next of kin or the postal system. Fatal CHD was confirmed by an examination of hospital or autopsy records, by the listing of CHD as the cause of death on the death certificate, if CHD was the underlying and most plausible cause, and if evidence of previous CHD was available.

The study protocol was approved by the institutional review board of the Brigham and Women's Hospital and the Human Subjects Committee Review Board of Harvard School of Public Health.

### Measurements

Demographic, anthropometric, and lifestyle data were derived from questionnaires administered at blood draw (1990 in the NHS and 1994 in the HPFS), with missing information substituted from previous questionnaires. Body mass index (BMI) was calculated as the weight in kilograms divided by the square of the height in meters. Physical activity was expressed in terms of metabolic equivalent hours. Participants reported whether a physician had ever diagnosed them with diabetes or hypertension. The questionnaires and the validity and reproducibility of measurements have been described previously.^31^ Blood samples were collected in tubes treated with liquid sodium heparin (in NHS) or EDTA (in HPFS). The tubes were then placed on ice packs, stored in styrofoam containers, returned to our laboratory by overnight courier, centrifuged, and divided into aliquots for storage in liquid-nitrogen freezers (−130°C or colder). Immuno-affinity chromatography was conducted with affinity-purified anti-human apoC-III (Academy Biomedical Company, Inc., Houston, TX) to separate the plasma into fractions with and without apoC-III. Detailed methods have been published previously.^34^ Subsequently, apoC-III-bound and apoC-III-unbound fractions were ultracentrifuged to isolate the very low-density (*d*<1.006 g/mL), low-density (1.006<*d*<1.063 g/mL), and high-density (*d*>1.063 g/mL) lipoprotein particles. The concentration of apoC-III in the bound fraction of HDL was determined by ELISA, and cholesterol was measured in both HDL fractions enzymatically (Thermo Scientific, Waltham, MA). Liquid transfer for 96-well plate loading and ELISA dilutions were handled robotically with a Multiprobe II (Perkin Elmer, Waltham, MA) to minimize pipetting error. Both ELISA and lipids plates were read with a BioTek ELx808iu 96-well plate reader controlled by KCJunior software (BioTek, Winooski, VT). All assays were completed in triplicate and any sample with an intraassay coefficient of variation more than 15% was repeated. One sample had to be reassessed for HDL-C with apoC-III and 30 samples for the concentration of apoC-III in HDL. Each batch in a laboratory analysis included the matched case-control sets so that run-to-run variation in the analysis would not add imprecision to the differences between cases and controls. All laboratory personnel were blinded to the case-control status. The within-run average CVs were 8% for HDL-C without apoC-III, 13% for HDL-C with apoC-III, and 17% for apoC-III in HDL.

A small fraction of the cholesterol in the standard HDL *d*>1.063 fraction prepared by ultracentrifugation could be transported in very dense LDL particles coisolated with HDL. Sufficient plasma volumes were available in the HPFS to repeat the measurement of cholesterol in HDL after precipitation of any contaminating apoB lipoproteins by dextran sulfate and magnesium chloride. These measurements were used in the analyses presented in this report. In the NHS this direct measurement was only possible in a subsample of 24 women with sufficient plasma left. We used this subset with both cholesterol measures in addition to measurements of the apoB concentration to estimate the average cholesterol per apoB particle in the *d*>1.063 fraction. The amount of cholesterol that was estimated to be associated with apoB particles in the *d*>1.063 fraction was 0.010 mmol/L in the fraction with apoC-III and 0.007 mmol/L in the fraction without apoC-III, comprising 4% and 0.5% of the fractions, respectively. Subsequently we computed corrected HDL-C measures for the NHS cohort by subtracting the cholesterol that was associated with very dense LDL in each of the apoC-III compartments. These values were used in the analyses presented in this report. Sensitivity analyses showed that the associations for total HDL-C and HDL-C without apoC-III were similar before and after applying the correction, whereas the association for HDL-C with apoC-III was slightly weaker after the correction.

We performed additional testing of our measurements on a set of newly collected samples that were analyzed with the above described methods in fresh state and after 6 days of freezer storage. We did not see any difference in the obtained values for the concentration of HDL-C with and without apoC-III from samples before and after storage.

### Statistical Analysis

We performed the current analysis in all participants with complete information on the HDL-C measures. After excluding participants with at least one value missing, we also excluded cases and controls that had no matching control or case (Figure 1). Our final data sets consisted of 572 in NHS (286 case-control sets) and 699 in HPFS (348 case-control sets; three cases had two matched controls). We compared baseline characteristics of the cases and controls who were part of versus those who were excluded from the analysis and found no statistically significant differences (Table, Appendix).

Baseline characteristics of participants who developed CHD during follow-up and controls were assessed in each cohort. Multivariable-adjusted relationships between cardiovascular risk factors and HDL-C with and without apoC-III were evaluated using mixed linear regression with a random effect for each matched pair (PROC mixed). Cohort-specific quintiles of lipid parameters were calculated using the distributions in the controls. Relative risks (RR) and 95% confidence intervals (CIs) for CHD risk were estimated by the incidence rate ratios from conditional logistic regression analyses taking into account the matching factors (age, smoking, and time of blood draw).^33^ Potential confounders were selected on the basis of prior knowledge of their role as cardiovascular risk factors or on the basis of their association with our HDL-C exposures of interest. Because HDL-C with and without apoC-III sums to total HDL-C, all models simultaneously included the two fractions. *P* values represent test of linear trend across quintiles. When no violations of the linear assumptions were identified we also analyzed the RR according to one standard deviation (SD) difference in total HDL-C (0.6 mmol/L. For HDL-C without apoC-III we used 0.53 mmol/L and for HDL-C with apoC-III 0.07 mmol/L to represent their relative proportions.). We tested for slope heterogeneity of the two HDL subtypes with the null hypothesis of them being equal. In the absence of between study heterogeneity, estimates from the two cohorts were pooled by random effects meta-analysis. In sensitivity analyses, we compared the associations from analyses in strata of 5 years of follow-up. Analyses were performed using SAS 9 (SAS Institute Inc., Cary, NC).

## Results

The NHS women were slightly younger, more likely to smoke, more likely to self-report a history of diabetes and hyper-cholesterolemia, and consumed less alcohol, compared with the HPFS men (Table 1). Besides expected differences in LDL-C and triglycerides levels, cases also had lower levels of HDL-C without apoC-III. The proportion of HDL-C that had apoC-III was 14% in the NHS and 11% in the men. It was slightly higher in cases compared with controls in both sexes (*P*=0.07 in NHS and *P*=0.01 in HPFS). The mean apoC-III concentration in HDL did not differ according to case status.

**Table 1.** Baseline Characteristics of Women and Men in Whom Coronary Heart Disease Developed During Follow-Up and Matched Controls in the Nurses’ Health Study (NHS) and the Health Professionals Follow-Up Study (HPFS)*

	NHS—Women		HPFS—Men	
	
Variable	Cases (*n*=286)	Controls (*n*=286)	Cases (*n*=348)	Controls (*n*=351)
Mean age (SD), y	60.2 (6.5)	60.1 (6.5)	64.3 (8.5)	64.2 (8.5)

Mean BMI (SD), kg/m^2^	26.8 (5.7)	25.3 (4.1)	26.1 (3.3)	25.5 (3.5)

Current smoker, %	26.6	25.9	8.1	7.7

Mean alcohol intake (SD), g/d	4.2 (8.0)	5.4 (9.4)	10.6 (15.5)	12.5 (15.0)

Physical activity (MET h/wk)	17.4 (20.7)	20.1 (21.4)	35.0 (36.5)	37.0 (38.1)

Caucasian ethnicity, %	98.6	99.7	98.0	98.3

Postmenopausal, %	86.7	84.6	N/A	N/A

Estrogen replacement therapy, %	31.0	32.6	N/A	N/A

Family history of MI, %	20.3	14.3	44.3	33.9

Diabetes, %	15.4	5.9	8.1	3.7

Hypercholesterolemia, %	54.2	41.6	47.7	39.3

Hypertension, %	53.9	31.8	36.8	29.1

Lipid/apoliprotein concentrations				

Mean total cholesterol (SD), mmol/L	6.07 (1.20)	5.97 (1.22)	5.65 (0.77)	5.42 (0.96)

Median triglyceride level (IQR), mmol/L	1.2 (0.8–1.9)	1.0 (0.8–1.5)	1.3 (0.9–1.9)	1.1 (0.7–1.5)

Mean LDL-C (SD), mmol/L	3.84 (1.06)	3.69 (1.05)	3.50 (0.88)	3.26 (0.85)

Mean HDL-C (SD), mmol/L	1.76 (0.56)	1.88 (0.65)	1.21 (0.43)	1.26 (0.41)

Mean HDL-C without apoC-III (SD), mmol/L	1.52 (0.52)	1.64 (0.61)	1.07 (0.39)	1.12 (0.37)

Mean HDL-C with apoC-III (SD), mmol/L	0.25 (0.11)	0.24 (0.10)	0.14 (0.07)	0.14 (0.07)

Mean apoC-III in HDL (SD), g/L	0.12 (0.10)	0.12 (0.07)	0.12 (0.10)	0.11 (0.08)

* Matching criteria were: age, smoking, and date of blood sampling. Values are means ± SD of continuous covariates or percentages. Lipid and apolipoprotein measures are in SI units. To convert cholesterol concentrations from mmol/L to mg/dL, divide by 0.0259. To convert ApoC-III from g/L to mg/dL, divide by 0.01. To convert triglycerides from mmol/L to mg/dL, divide by 0.0113. Triglyceride levels were log-transformed before analysis and only reported in fasting participants (HPFS=65%, NHS=79%).

Diabetes, hypertension, and hypercholesterolemia were self-reported diagnosis before blood draw.

BMI indicates body mass index; MET, metabolic equivalent; MI, myocardial infarction;IQR, interquartile range

**Figure 2.**
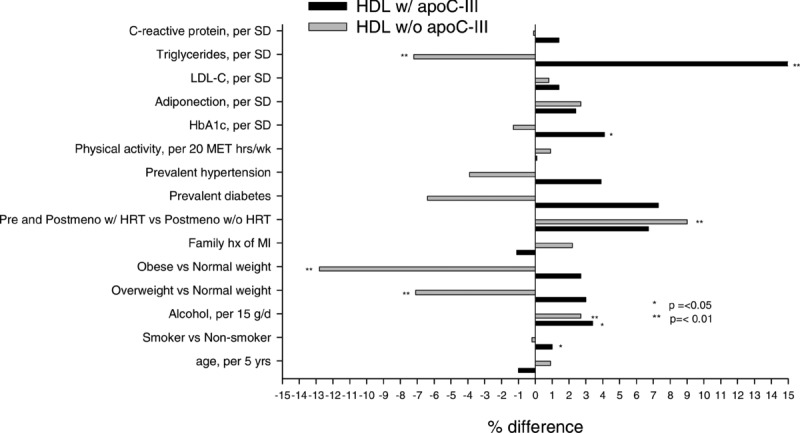
Multivariate-adjusted percent differences in levels of HDL-C with and without apoC-III according to demographic, lifestyle, and clinical factors in the NHS and the HPFS. Multivariate-adjusted regression analyses for the association of each factor with the log-transformed HDL levels, adjusted for each variable simultaneously. Biomarkers were evaluated one at a time in multivariable-adjusted models. Mixed-effect models to take matching into account. Estimates from the two cohorts were pooled using random-effects meta-analysis. No *P* values for test of between study heterogeneity were lower than 0.5. All variables assessed at baseline (time of blood draw).

We analyzed whether lifestyle factors and biomarkers known to be associated with cardiovascular risk were differentially associated with levels of HDL-C with and without apoC-III. Compared with normal weight, overweight and obesity was associated with 7% (*P*=0.0002) and 12% (*P*=0.01) lower levels of HDL-C without apoC-III, whereas elevated body mass index was statistically insignificantly associated with higher levels of HDL-C with apoC-III (Figure 2). Alcohol was associated with approximately 3% (*P*=0.04 and 0.01, respectively) higher levels of both HDL-C types per each 15 g/day. Smokers had 1% (*P*=0.04) higher levels of HDL-C with apoC-III compared with nonsmokers. Levels of HDL-C without apoC-III were 9% (*P*=0.02) higher in premenopausal women and postmenopausal women who received hormone replacement than postmenopausal women who did not take hormones. We evaluated cardiovascular biomarkers in separate models and found that higher triglyceride levels were associated wit h higher levels of HDL-C with apoC-III, and lower levels of HDL-C without apoC-III (both *P* = 0.001).

### HDL-C With and Without ApoC-III and Risk for Future CHD

**Figure 3.**
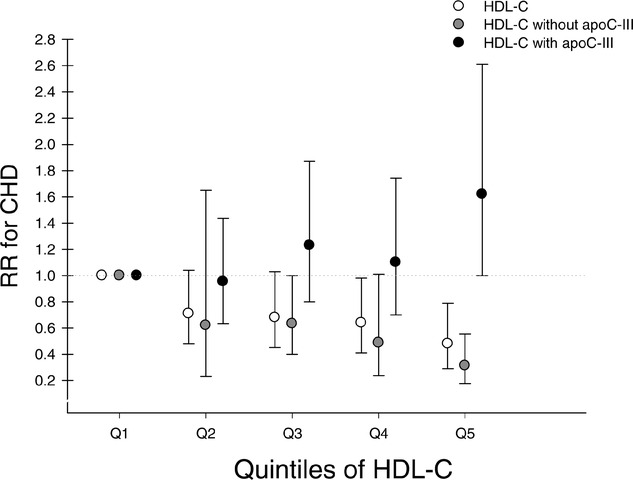
Multivariate-adjusted RRs for CHD according to quintiles of total HDL-C, HDL-C with and without apoC-III in the combined NHS and HPFS. RRs are incidence rate ratios (IRR) obtained from conditional logistic regression models. Multivariate model takes into account age and smoking due to matching. HDL-C with and without apoC-III are simultaneously included. NHS and HPFS data were combined using random effects meta-analyses. No *P* values for test of between study heterogeneity were lower than 0.5. Error bars indicate 95% confidence interval. Adjusted for alcohol, body mass index, self-reported diagnosis of hypertension before blood draw, postmenopausal status, and hormones in NHS. The *P* for linear trend across quintiles: total HDL-C=0.005; for HDL-C without apoC-III=0.007; HDL-C with apoC-III=0.02. *P* for test of difference in slope between HDL-C with and without apoC-III=0.02.

Total HDL-C was inversely associated with risk of CHD in both NHS and HPFS (Table 2). An even stronger inverse association was observed across quintiles of HDL-C without apoC-III in both cohorts. The associations appeared slightly stronger in the NHS women than the HPFS men, but the tests of between study heterogeneity for the associations were not significant (*P*>0.5). When the cohort-specific results from multivariable analyses that adjusted for important demographics and lifestyle factors were combined, the RR across extreme quintiles of HDL-C without apoC-III was 0.31 (95% CI, 0.18–0.55) in the combined cohort (Figure 3). Each SD increase (0.53 mmol/L) predicted a RR of 0.66 (95% CI, 0.53–0.83; Table 3). In contrast, higher levels of HDL-C with apoC-III were not inversely associated with the risk of CHD. In the combined cohorts, the RR for quintile 5 versus 1 was 1.62 (CI, 1.00–2.61). Each SD increase in HDL-C with apoC-III (0.07 mmol/L) was associated with an 18% (95% CI, 3–34%) higher risk of future CHD. The slopes of the regression coefficients for the two HDL-C subtypes were statistically significantly different (*P*=0.02). Additional adjustment for triglycerides and apoB attenuated the risk estimates for HDL-C with apoC-III but the test of heterogeneity for slopes remained statistically significant (*P*=0.05). After additional adjustment for the potential intermediate exposure, diabetes, the HDL-C with apoC-III was no longer associated with the risk of CHD (RR per SD=1.02 [95% CI, 0.88–1.18]), whereas an inverse trend remained for HDL-C without apoC-III (RR per SD=0.79 [95% CI, 0.62–1.01]; Table 3).

**Table 2.** Incidence Rate Ratios (IRR) and 95% confidence intervals of CHD According to Quintiles of Total HDL-C, HDL-C Without ApoC-III and HDL-C With ApoC-III in the Nurses’ Health Study (NHS) and the Health Professionals Follow-Up Study (HPFS)

	Q1	Q2	Q3	Q4	Q5	*P* trend
Total HDL-C						

*NHS* (median, range)	1.17 (0.32–1.37)	1.53 (1.38–1.67)	1.81 (1.68–1.97)	2.12 (1.98–2.36)	2.68 (2.37–4.20)	

Unadjusted	*1.0 (ref)*	0.86 (0.51–1.45)	0.67 (0.37–1.21)	0.70 (0.39–1.26)	0.32 (0.15–0.65)	0.003

Multivariate	*1.0 (ref)*	0.88 (0.50–1.54)	0.68 (0.36–1.30)	0.79 (0.41–1.51)	0.41 (0.18–0.92)	0.05

*HPFS* (median, range)	0.81 (0.22–0.93*)*	1.04 (0.93–1.14)	1.23 (1.14–1.33)	1.42 (1.33–1.58)	1.79 (1.58–3.59)	

Unadjusted	*1.0 (ref)*	0.58 (0.36–0.94)	0.64 (0.38–1.07)	0.48 (0.27–0.84)	0.46 (0.25–0.85)	0.02

Multivariate	*1.0 (ref)*	0.59 (0.36–0.97)	0.68 (0.40–1.16)	0.54 (0.30–0.96)	0.53 (0.28–1.01)	0.07

HDL-C without ApoC-III						

*NHS* (median, range)	0.95 (0.03–1.14)	1.32 (1.14–1.46)	1.59 (1.46–1.75)	1.88 (1.75–2.11)	2.39 (2.12–3.70)	

Unadjusted	*1.0 (ref)*	0.90 (0.49–1.65)	0.57 (0.29–1.11)	0.58 (0.28–1.22)	0.20 (0.09–0.49)	0.0001

Multivariate	*1.0 (ref)*	1.03 (0.54–1.95)	0.68 (0.33–1.42)	0.74 (0.33–1.65)	0.30 (0.12–0.77)	0.006

*HPFS* (median, range)	0.71 (0.19–0.83)	0.90 (0.83–1.00)	1.09 (1.00–1.18)	1.27 (1.18–1.42)	1.61 (1.42–3.34)	

Unadjusted	*1.0 (ref)*	0.39 (0.23–0.68)	0.52 (0.30–0.93)	0.32 (0.17–0.59)	0.26 (0.13–0.53)	0.006

Multivariate	*1.0 (ref)*	0.38 (0.21–0.67)	0.61 (0.34–1.09)	0.35 (0.19–0.66)	0.32 (0.16–0.66)	0.03

HDL-C with apoC-III						

*NHS* (median, range)	0.12 (0.01–0.16)	0.16 (0.15–0.20)	0.23 (0.20–0.26)	0.28 (0.26–0.32)	0.39 (0.33–0.59)	

Unadjusted	*1.0 (ref)*	0.86 (0.47–1.56)	1.12 (0.62–2.04)	1.09 (0.57–2.09)	1.58 (0.79–3.16)	0.08

Multivariate	*1.0 (ref)*	0.78 (0.41–1.46)	1.12 (0.60–2.11)	1.00 (0.51–1.99)	1.44 (0.69–3.01)	0.14

*HPFS* (median, range)	0.07 (0.01–0.08)	0.10 (0.09–0.11)	0.13 (0.12–0.14)	0.16 (0.14–0.18)	0.22 (0.18–0.51)	

Unadjusted	*1.0 (ref)*	1.15 (0.69–1.93)	1.34 (0.80–2.32)	1.19 (0.66–2.15)	1.77 (0.96–3.24)	0.07

Multivariate	*1.0 (ref)*	1.10 (0.64–1.89)	1.32 (0.74–2.33)	1.19 (0.65–2.19)	1.76 (0.94–3.31)	0.08

Incidence rate ratios (IRR) obtained from conditional logistic regression models. Unadjusted model takes into account age and smoking (due to matching). Multivariate model includes: alcohol, body mass index, self-reported diagnosis of hypertension before blood draw, and postmenopausal status and hormones in NHS only. HDL with and without apoC-III are simultaneously included in all models. *P* trend is the test for linear trend across quintiles.

**Table 3.** Incidence Rate Ratios (IRR) and 95% confidence intervals of CHD According to Continuous Measures of Total HDL-C (per 0.60 mmol/L), HDL-C Without ApoC-III (per 0.53 mmol/L), and HDL-C with ApoC-III (per 0.07 mmol/L) in the Nurses’ Health Study (NHS) and the Health Professionals Follow-Up Study (HPFS)

	NHS		HPFS			Together	
	Per SD	*P*	Per SD	*P*	*P* het	Per SD	*P*
Total HDL-C							

Unadjusted	0.69 (0.54–0.87)	0.002	0.70 (0.52–0.95)	0.02	0.9	0.69 (0.58–0.83)	0.0001

Adjusted	0.77 (0.59–1.01)	0.06	0.79 (0.57–1.10)	0.2	0.9	0.78 (0.63–0.96)	0.02

+ triglycerides and apoB	0.81 (0.61–1.07)	0.14	0.85 (0.61–1.18)	0.3	0.8	0.83 (0.68–1.01)	0.06

+ diabetes	0.80 (0.60–1.05)	0.11	0.86 (0.61–1.20)	0.4	0.7	0.82 (0.66–1.02)	0.07

HDL without ApoC-III							

Unadjusted	0.55 (0.42–0.73)	0.0001	0.61 (0.44–0.85)	0.003	0.6	0.58 (0.47–0.71)	<0.0001

Adjusted	0.64 (0.47–0.87)	0.004	0.70 (0.50–0.97)	0.03	0.7	0.66 (0.53–0.83)	0.0001

+ triglycerides and apoB	0.70 (0.50–0.98)	0.04	0.85 (0.60–1.21)	0.4	0.4	0.77 (0.60–0.98)	0.03

+ diabetes	0.73 (0.82–1.02)	0.07	0.87 (0.61–1.25)	0.4	0.5	0.79 (0.62–1.01)	0.06

HDL with ApoC-III							

Unadjusted	1.16 (1.00–1.35)	0.05	1.20 (0.97–1.47)	0.09	0.8	1.17 (1.04–1.32)	0.01

Adjusted	1.16 (0.99–1.35)	0.07	1.21 (0.98–1.51)	0.08	0.7	1.18 (1.03–1.34)	0.01

+ triglycerides and apoB	1.10 (0.93–1.30)	0.3	1.00 (0.78–1.29)	0.9	0.6	1.07 (0.93–1.23)	0.4

+ diabetes	1.04 (0.87–1.24)	0.6	0.97 (0.76–1.25)	0.8	0.7	1.02 (0.88–1.18)	0.8

Incidence rate ratios (IRR) obtained from conditional logistic regression models.Unadjusted model takes into account age and smoking (due to matching). Multivariate model includes: alcohol, body mass index, self-reported diagnosis of hypertension before blood draw, and postmenopausal status and hormones in NHS only. HDL with and without apoC-III are simultaneously included in all models. The NHS and HPFS data were combined using random effects meta-analyses. *P* het=*P* for test of between study heterogeneity

### Investigation of Potential Effect-Modifiers

We addressed whether the observed associations for HDL-C with and without apoC-III would be modified by important CVD risk factors. We tested for the potential effect-modification by median triglycerides and did not observe any (*P* for interaction HDL-C with and without apoC-III both >0.1). There was no evidence of effect-modification by hypercholesterolemia (both *P* for interaction >0.1). We had few participants who reported use of cholesterol-lowering drugs at blood draw in 1990/1994, but our results were not appreciably different when restricted to participants who did not take cholesterol-lowering drugs. Finally, the majority of the women were postmenopausal, and 33% of them reported use of estrogen replacement therapy at baseline. In analyses among postmenopausal women alone we observed very similar trends for HDL-C with and without apoC-III in strata according to use of estrogen replacement therapy (both *P* for interaction>0.1).

### Concentration of apoC-III in HDL and Risk for Future CHD

The concentration of apoC-III in HDL tended toward a direct association with risk of CHD in both cohorts. The RR for highest versus lowest quintile in a multivariable-adjusted model with additional adjustment for HDL-C was 1.42 (0.79 to 2.57), but the trend across quintiles was also insignificant (*P* trend=0.2; Table 4).

**Table 4.** Incidence Rate Ratios (IRR) and 95% confidence intervals for CHD According Quintiles of ApoC-III in HDL in the Nurses’ Health Study (NHS) and the Health Professionals Follow-Up Study (HPFS)

	Q1	Q2	Q3	Q4	Q5	*P* trend
*NHS*						

Median (range)	0.03 (0.003–0.05)	0.07 (0.05–0.09)	0.10 (0.09–0.12)	0.15 (0.12–0.17)	0.22 (0.17–1.25)	

Unadjusted	*1.0 (ref)*	1.55 (0.81–2.94)	1.19 (0.57–2.46)	1.20 (0.55–2.69)	1.51 (0.62–3.69)	0.6

Multivariate	*1.0 (ref)*	1.52 (0.76–3.04)	1.11 (0.51–2.41)	1.06 (0.46–2.43)	1.14 (0.44–2.97)	0.8

*HPFS*						

Median (range)	0.04 (0.0004–0.06)	0.07 (0.05–0.08)	0.09 (0.08–0.11)	0.13 (0.11–0.16)	0.21 (0.16–0.72)	

Unadjusted	*1.0 (ref)*	0.95 (0.57–1.59)	0.76 (0.43–1.36)	1.58 (0.89–2.80)	1.67 (0.82–3.41)	0.04

Multivariate	*1.0 (ref)*	1.03 (0.60–1.76)	0.77 (0.42–1.40)	1.60 (0.88–2.92)	1.63 (0.77–3.46)	0.07

*Together*						

Unadjusted	*1.0 (ref)*	1.17 (0.73–1.86)	0.91 (0.58–1.42)	1.44 (0.91–2.28)	1.60 (0.92–2.80)	0.07

Multivariate	*1.0 (ref)*	1.29 (0.78–1.82)	0.88 (0.55–1.42)	1.39 (0.85–2.26)	1.42 (0.79–2.57)	0.2

Incidence rate ratios (IRR) obtained from conditional regression models. Unadjusted model takes into account age and smoking (due to matching). Multivariate model includes: alcohol, body mass index, total HDL-C, self-reported diagnosis of hypertension before blood draw, and postmenopausal status and hormones in NHS only. The NHS and HPFS data were combined using random effects meta-analyses. No *P* values for test of between study heterogeneity were lower than 0.5. *P* trend is the test for linear trend across quintiles.

## Discussion

The controversies in establishing the role of HDL in atherosclerosis may be due in part to the lack of specificity in the measurements of HDL-C. In two independent prospective studies of generally healthy middle-aged men and women, we found that HDL is composed of two populations having opposite associations with CHD. The major HDL-C type lacking apoC-III has the expected protective association with CHD, whereas the small subfraction of HDL-C that has apoC-III present on its surface (≈13%) tended to be associated with a higher risk of future CHD.

Investigations of the metabolic heterogeneity of lipid particles are potentially valuable to improve understanding of the atheroprotective or nonprotective effects of HDL. Although previous large-scale studies have evaluated HDL subpopulations separated by size,^21^ whether such measurements improve cardiovascular risk prediction remains uncertain as the findings have been inconsistent.^15,35^ Other more experimental subclassifications include the effect of HDL on cholesterol efflux or anti-inflammatory activities of HDL.^36^ These new experimental assays are of scientific interest and suggest that the measure of total HDL-C may be diluted due to a mixing of cholesterol distributed in both anti- and proatherogenic HDL particles. However, so far, the concept and understanding of what makes a dysfunctional or even proinflammatory HDL subtype remains elusive.^14,37^ Our data suggest that apoC-III may confer atherogenic properties to HDL that potentially could overcome other beneficial components. We observed that the apoC-III concentration in HDL was not significantly associated with CHD, indicating that the concentration of HDL-C with *any* apoC-III may be more relevant to the risk of CHD than how much apoC-IIII is in the HDL. Previous studies have only addressed the latter question. In the CLAS trial, the concentration of apoC-III in HDL was inversely associated with the progression of CAD in the drug-treated group of CAD patients only,^38^ whereas a direct, but not statistically significant, association with CHD and re-current events was reported in two larger studies.^26,39^ These studies were either cross sectional^39^ or conducted in a patient population with existing CVD.^26^ Because the concentration of apoC-III may be affected by disease status, it may be particularly important to study this in a prospective setting in populations that did not have clinical CVD at baseline. For the metabolism of entire lipoprotein particles, it is likely that the presence (if any) versus absence of apoC-III may determine the downstream interactions with receptors and enzymes.^22,40^. Kawakami et al^25^ reported that HDL without apoC-III, but not HDL with apoC-III, limits the proinflammatory adhesion of human monocytes to endothelial cells. ApoC-III also plays an important role in the catabolism of triglyceride-rich lipoproteins through the inhibition of clearance of plasma VLDL and LDL by the liver.^41–43^ It is possible that apoC-III functions similarly in HDL circulating in blood, impairing delivery of HDL-C to the liver. However, it remains a possibility that apoC-III is a marker for other attributes of HDL that are related to atherosclerosis.

Lifestyle factors may modulate the distribution of cholesterol within the two HDL fractions. We found that alcohol intake was similarly associated with both HDL-C subfractions, whereas body weight and estrogens were only associated with HDL-C without apoC-III. Other unmeasured confounders cannot be excluded.

We used the concentration of cholesterol in HDL as our measure of HDL. It is possible that our results could be refined by assessing alternative measures, such as the concentration of apoA-I, the major apolipoprotein component of HDL, instead. It is among our study limitations that we only had one assessment of the lipid subfractions. Thus, our findings cannot determine whether changes in the proportions of cholesterol transported in HDL particles with and without apoC-III are causally related to risk of CHD. Furthermore, the immunoaffinity chromatography approach is a lengthy procedure and nondifferential measurement error might have diluted our relative risk estimates. Earlier studies of apoC-III in lipoprotein fractions have reported concern about redistribution of apoC-III from apoB-containing lipoproteins to HDL during storage.^44^ We did not detect instability in our measures of HDL-C with (*any*) apoC-III and HDL-C without apoC-III in our own assessment of samples that were analyzed both fresh and after freezer storage. Use of different methodologies for the separation of the apoC-III—containing lipoprotein fractions may be one explanation.

Although our nested case-control study was prospective in nature, undiagnosed illness at baseline might create a spurious association. We compared the associations from analyses in strata of 5 years of follow-up. The results were similar to those presented here both when cases that occurred during the first five and subsequent years of follow-up were considered (data not shown).

In conclusion, we found that HDL-C with and without apoC-III showed opposite associations with the risk of CHD in prospective studies of apparently healthy men and women. On adjustment for a proatherogenic lipid profile and diabetes, HDL-C with apoC-III was no longer associated with risk of CHD, but there was no evidence for an inverse association. Our findings highlight that HDL comprises a group of particles that may be more or less closely linked with atherosclerosis. HDL that has apoC-III may represent a dysfunctional HDL lacking its cardioprotective function. This may also have implications for future development novel therapeutic interventions aimed at HDL elevation, as the cardioprotective benefits may differ depending on the affected HDL subfraction.
